# International travel as source of a hospital outbreak with an unusual meticillin-resistant *Staphylococcus aureus* clonal complex 398, Denmark, 2016

**DOI:** 10.2807/1560-7917.ES.2019.24.42.1800680

**Published:** 2019-10-17

**Authors:** Jens Kjølseth Møller, Anders Rhod Larsen, Claus Østergaard, Camilla Holten Møller, Mette Assenholm Kristensen, Jesper Larsen

**Affiliations:** 1 Department of Clinical Microbiology, Lillebælt Hospital, Vejle, Denmark; 2 Institute of Regional Health Research, Faculty of Health Sciences, University of Southern Denmark, Odense, Denmark; 3 Department of Bacteria, Parasites & Fungi, Statens Serum Institut, Copenhagen, Denmark; 4 Department of Infectious Disease Epidemiology & Prevention, Statens Serum Institut, Copenhagen, Denmark

**Keywords:** meticillin-resistant Staphylococcus aureus, MRSA, Panton-Valentine leukocidin, PVL, hospital outbreak, surveillance, travel, epidemiology

## Abstract

In May 2016, an unusual outbreak with the Panton-Valentine leukocidin-positive human variant of meticillin-resistant *Staphylococcus aureus* (MRSA) clonal complex 398 occurred among mothers and infants in the maternity unit of a Danish hospital. MRSA sharing genotypic and phenotypic characteristics was confirmed in 36 cases, including 26 patients, nine household members and a healthcare worker (HCW) who had contact with all the patients. The national MRSA database contained 37 seemingly unlinked MRSA cases whose isolates shared the same genotypic and phenotypic characteristics as the outbreak strain. Whole genome sequencing showed that three of these isolates clustered together with the 36 outbreak isolates, suggesting spread outside the hospital. The HCW and 21 of 37 cases from the national MRSA database had links to south-eastern Asia, where the outbreak strain is endemic. These findings suggest that the HCW acquired the outbreak strain while travelling in south-eastern Asia and then introduced it into the hospital; from there, it spread within the patients’ households and into the community. Screening of travellers returning from countries with high levels of MRSA could be an important intervention to prevent spread of these bacteria into hospitals via patients or HCWs.

## Background

Meticillin-resistant *Staphylococcus aureus* (MRSA) is one of the world’s leading causes of infections in hospitals and other healthcare facilities. In 2015, MRSA accounted for an estimated 148,727 infections, 68% of which were healthcare associated, and 7,049 deaths in the European Union and European Economic Area (EU/EEA) [1].

Denmark is considered a low prevalence country for MRSA. According to the latest report from the European Antimicrobial Resistance Surveillance Network (EARS-Net), MRSA accounted for only 2.0% of *S*. *aureus* bacteraemia cases in Denmark, compared with 13.7% across Europe [2]. Nonetheless, the number of infections caused by imported, community-associated and livestock-associated (LA) MRSA clones is increasing [3]. Large hospital outbreaks with MRSA are uncommon in Denmark, with the exception of an epidemic MRSA-15 clone (EMRSA-15) outbreak that affected 440 individuals in two hospitals in Vejle County from 2002 to 2005 [4]. In 2016, MRSA only caused an estimated 30 hospital-acquired infections (HAIs), compared with 176 infections among other individuals who were recently exposed to a healthcare setting, 325 imported infections, 607 community-acquired infections and 218 infections with the LA variant of MRSA clonal complex 398 (LA-MRSA CC398) [3].

In 2006, the Danish health authorities made MRSA a notifiable organism and adopted a national multifaceted programme to prevent the introduction of MRSA into healthcare settings from expanding community and livestock reservoirs. The programme consists of several elements, including development and maintenance of a national surveillance system, a restrictive antimicrobial policy and a set of infection control guidelines. These guidelines recommend targeted screening of patients with predefined risk factors for MRSA acquisition at hospital admission, as well as decolonisation and isolation of carriers [5]. As part of the programme, the local clinical microbiology departments perform *S*. *aureus* identification and antimicrobial susceptibility testing, then submit confirmed MRSA isolates to the National Reference Laboratory for Antimicrobial Resistance (NRLAR) at Statens Serum Institut (SSI) for further genotypic and phenotypic characterisation. Within a week, the local clinical microbiology departments receive a report containing strain typing results for each submitted MRSA isolate, including the *spa* type and/or the clonal complex. Strain typing results and notification forms with patient information are stored in the national MRSA repository and database, which are maintained by NRLAR for surveillance purposes.

## Outbreak detection

On 3 May 2016, the Department of Clinical Microbiology at Lillebælt Hospital became aware of a possible outbreak after MRSA was isolated from two women’s breast abscesses on 7 and 29 April 2016, respectively. Both had given birth in the maternity unit at Kolding Hospital in March 2016. MALDI-TOF mass spectrometry (MALDI-TOF MS) revealed that the two MRSA isolates belonged to a unique subgroup, previously termed A8.3 [6], which is related to yet distinct from the LA-MRSA CC398 MALDI-TOF MS subgroups prevalent in the hospital’s catchment area. Comparison of the antibiograms showed that the isolates had identical profiles, as they were all resistant to penicillin, cefoxitin, tetracycline, erythromycin and clindamycin. NRLAR was then contacted to obtain further genotypic and phenotypic characteristics of the two MRSA isolates, which showed that they belonged to CC398, had *spa* type t034 and carried *mecA*, *scn* and *lukF-PV*, which together with *lukS-PV* encodes Panton-Valentine leukocidin (PVL). The presence of *lukF-PV* suggested that the isolates belonged to the human variant of MRSA CC398, which was considered extremely rare in Denmark at that time [7]. This further convinced the infection control team that the two cases had acquired the strain from a common source during their stay in the maternity ward, and a formal outbreak investigation was therefore opened. Here we describe the main findings of the outbreak investigation and consider some of the many challenges associated with outbreak prevention, detection and management.

## Methods

### Ethics statement

This study was a collaboration between the Department of Clinical Microbiology at Lillebælt Hospital and NRLAR at SSI. Collection and use of data were approved by the Danish Data Protection Agency (protocol number 2001–14–0021).

### Setting

Lillebælt Hospital serves a catchment area of 300,000 inhabitants and has about 700 beds and 200,000 occupied-bed-days per year. The trust comprises three hospitals in Kolding, Middelfart and Vejle, in the Region of Southern Denmark. The maternity unit where the outbreak occurred is located at Kolding Hospital, where an average of 3,200 births take place each year.

### Case definition

On 4 May 2016, an outbreak group consisting of representatives from the maternity unit and the infection control team at the Department of Clinical Microbiology initiated a formal investigation. Possible cases were defined as mothers who had undergone caesarean delivery or other birth-related surgical procedures in the maternity unit at Kolding Hospital in early May 2016. This definition was changed on 10 May 2016 to include all mothers, infants and staff members who had worked or stayed in the maternity unit between 1 January and 30 June 2016. Probable cases were defined as all individuals who had worked or stayed in the maternity unit between 1 January and 30 June 2016 and all their household contacts who were found to be colonised or infected with MRSA CC398 of MALDI-TOF MS subgroup A8.3 between 1 January 2016 and 28 February 2017. Confirmed cases were defined as probable cases whose isolates belonged to the outbreak cluster, as determined by phylogenetic analysis of high-quality single nucleotide polymorphisms (SNPs) in the core genome.

### Outbreak investigation

The outbreak investigation comprised: (i) immediate risk assessment of procedures involving the use and storage of breastfeeding equipment, as well as postoperative management of mothers who underwent caesarean delivery or other delivery-related surgical procedures; (ii) environmental sampling of common contact points in the delivery room, operating room and milk kitchen of the maternity unit, e.g. soap dispensers and handles of drawers, cabinets and doors (n = 32); (iii) interviews with hospital staff members; (iv) screening of all mothers and infants (n = 60), as well as all hospital staff members (n = 88), who were present in the maternity unit or the neonatal unit on 10 May 2016; (v) contacting all mothers who had stayed in the maternity unit between 1 April and 9 May 2016 (n = 177) to promote screening of the entire household by their general practitioner and (vi) retrospective and prospective review of the local MRSA database Laboratorieovervågning af antibiotikaresistens (LIVA) to identify individuals who had tested positive for MRSA CC398 of MALDI-TOF MS subgroup A8.3 outside the hospital between January 2016 and February 2017, including those who were contacted by the infection control team.

LIVA is a web-based database application for real-time surveillance of MRSA in the Region of Southern Denmark and contains information from the following sources: (i) demographic data from the Danish Civil Registration System (CPR); (ii) local clinical microbiology laboratory-based results of MRSA cultures from screening swabs and clinical samples, positive or negative; (iii) local and NRLAR-based strain typing results for laboratory-confirmed MRSA isolates, including MALDI-TOF MS subgroup profiles, *spa* types and clonal complexes; and (iv) epidemiological and clinical data for all MRSA-positive cases collected by clinical microbiologists and infection control nurses.

During the outbreak investigation, the following data were assessed for each MRSA-positive case: sex, age, country of birth, residential address, specimen source (screening swab or clinical sample), infection type, hospitalisation dates, MALDI-TOF MS subgroup profile, *spa* type and clonal complex.

### Sample collection and laboratory investigations

Clinical samples were obtained from patients with active infection and were cultured directly on 5% blood agar and incubated for 24–48 hours at 35 °C. Healthy mothers, infants and hospital staff members were screened for MRSA by swabbing the nose and throat. Screening swabs and environmental swabs collected in the maternity unit were enriched for at least 24 hours at 35 °C in Contrast MRSA Broth (Oxoid, Basingstoke, United Kingdom). A 10 μl loopful of broth from positive cultures (colour change from red) was plated onto a CHROMID MRSA agar plate (bioMérieux, Marcy l'Etoile, France) and incubated for 24 hours at 35 °C, whereafter one presumptive MRSA colony (green colour) was subcultured on 5% blood agar for 24 hours at 35 °C. Colonies with morphological characteristics of *S*. *aureus* on 5% blood agar underwent antimicrobial susceptibility testing against penicillin, cefoxitin, tetracycline, erythromycin, clindamycin, gentamicin, rifampicin, fusidic acid, mupirocin and linezolid using the disk diffusion method in accordance with the European Committee on Antimicrobial Susceptibility Testing (EUCAST) guidelines [8]. Presumptive MRSA colonies growing within the inhibition zone of the cefoxitin disks were subjected to MALDI-TOF MS for species identification and subgrouping of MRSA isolates using the microflex LT/SH instrument (Bruker, Bremen, Germany), as described elsewhere [9], and investigated for the presence of PBP2a using a commercial latex agglutination kit, Slidex MRSA detection (bioMérieux).

All MRSA isolates were routinely submitted to NRLAR, where they were investigated for the presence of *spa*, *mecA*, *scn*, *lukF-PV* and the *S*. *aureus* CC398-specific *sau1-hsdS1* variant using a multiplex PCR assay. Methodological details are provided in Supplement S1. *spa*-typing was performed as described previously [10].

### Identification of other Danish cases between January 2006 and April 2017

The national MRSA database was reviewed to identify other Danish cases of PVL-positive MRSA CC398 between 1 January 2006 and 30 April 2017. The following data were assessed for each case: sex, age, country of birth, residential address, travel history, specimen source (screening swab or clinical sample), infection type and hospitalisation dates.

### Whole genome sequencing and bioinformatics analyses

Whole genome sequencing and bioinformatics analyses were used to study the relationship of the PVL-positive MRSA CC398 isolates identified in this study. Methodological details are provided in Supplement S1.

### Data access

The whole genome sequence data from this study have been submitted to the National Center for Biotechnology Information (NCBI) Sequence Read Archive (SRA) under BioProject PRJNA508272.

## Results

### Outbreak cases identified in the maternity unit

**Figure 1:**
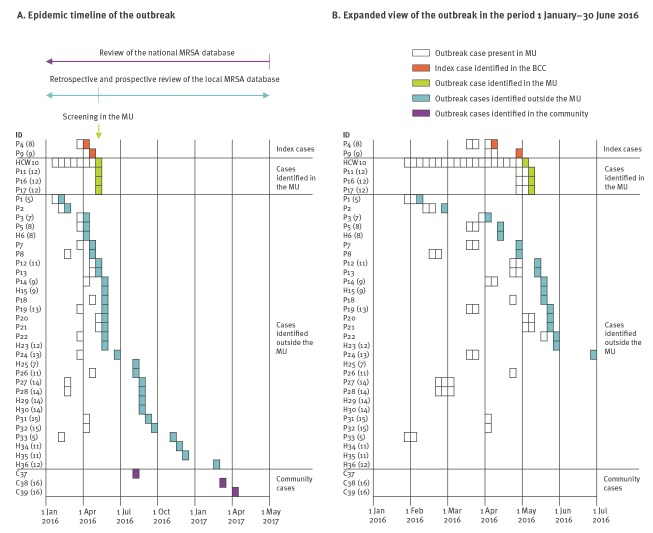
Timeline of the *Staphylococcus aureus* clonal complex 398 hospital outbreak, Kolding, Denmark, 1 January 2016–30 April 2017 (n = 39)

BCC: breast care centre; C: community case; H: household contact; HCW: healthcare worker; P: patient; MU: maternity unit.

On 5 May 2016, a healthcare worker (HCW) (outbreak case 10) who had been in contact with the two index cases (outbreak cases 4 and 9) screened positive for MRSA. The HCW had also been in contact with mothers whose children were transferred to the neonatal unit at the same hospital. This prompted the infection control team to include the neonatal unit in the outbreak investigation. Five days later, on 10 May 2016, all 23 mothers and 16 infants in the maternity unit were screened for MRSA carriage, as were eight mothers and 13 infants in the neonatal unit and 88 hospital staff members. This led to the identification of three probable outbreak cases in the maternity unit, a mother and her newborn twins (outbreak cases 11, 16 and 17) (Figure 1 and Table 1). All three cases had already been discharged when the results of their MRSA status became available and, therefore, isolation precautions were not needed. The isolates from these cases and the HCW belonged to the same unique MALDI-TOF MS subgroup A8.3 and antibiogram profile as the isolates from the two index cases. All other hospital staff members, mothers and infants in the maternity and neonatal units screened negative.

Cases were categorised as healthcare workers, patients, household contacts or community cases, and were assigned case numbers according to the sampling date.

Household cluster IDs are indicated in parentheses.

**Table 1:** Description of MRSA cases and isolates identified during hospital outbreak investigation, Kolding, Denmark, 2016–2017 (n = 36)

Outbreak case number^a^	Isolate ID	Case description	Household cluster	Hospitalisation dates in the maternity unit^b^ (month and year)	Sampling date	Sampling place	Specimen source
**Index cases**							
4	216065	Mother	8	Mar 2016	Apr 2016	BCC	Breast abscess
9	216913	Mother	9	Apr 2016	Apr 2016	BCC	Breast abscess
**Cases identified in the MU**							
10	217161	HCW in MU	NA	NA	May 2016	MU	Screening^c^
11	217479	Mother	12	May 2016	May 2016	MU	Screening^d^
16	217637	Infant	12	May 2016	May 2016	MU	Conjunctivitis, scalp pustules
17	217639	Infant	12	May 2016	May 2016	MU	Scalp pustules
**Cases identified outside the MU**							
1	213707	Mother	5	Feb 2016	Feb 2016	GP	Postoperative wound
2	214441	Infant	NA	Feb 2016	Feb 2016	HCF	Scalp pustules
3	216051	Infant	7	Mar 2016	Apr 2016	HCF	Conjunctivitis
5	216247	Infant	8	Mar 2016	Apr 2016	GP	Screening
6	216245	Father	8	Not hospitalised	Apr 2016	GP	Screening
7	216863	Mother	NA	Mar 2016	Apr 2016	GP	Axillary abscess
8	216867	Infant	NA	Feb 2016	Apr 2016	GP	Conjunctivitis
12	217389	Infant	11	Apr 2016	May 2016	GP	Screening
13	217491	Mother	NA	Apr 2016	May 2016	GP	Breast abscess, postoperative wound
14	217573	Infant	9	Apr 2016	May 2016	GP	Screening
15	217681	Grandparent	9	Not hospitalised	May 2016	GP	Screening
18	217683	Infant	NA	Apr 2016	May 2016	HCF	Screening
19	217817	Mother	13	Mar 2016	May 2016	GP	Breast abscess
20	217831	Infant	NA	May 2016	May 2016	GP	Screening
21	218157	Infant	NA	May 2016	May 2016	GP	Bulla (gluteal region)
22	218159	Mother	NA	Mar 2016	May 2016	GP	Axillary abscess
23	219585	Sibling	12	Not hospitalised	May 2016	GP	Wound (leg)
24	219599	Infant	13	Mar 2016	Jun 2016	GP	Screening
25	220909	Father	7	Not hospitalised	Aug 2016	GP	Wound (leg)
26	220981	Mother	11	Apr 2016	Aug 2016	BCC	Breast abscess
27	221423	Infant	14	Feb/Mar 2016	Aug 2016	GP	Eczema
28	221793	Mother	14	Feb/Mar 2016	Aug 2016	GP	Screening
29	221789	Sibling	14	Not hospitalised	Aug 2016	GP	Screening
30	221735	Father	14	Not hospitalised	Aug 2016	GP	Screening
31	222407	Mother	15	Apr 2016	Sep 2016	GP	Skin abscess (mosquito bite)
32	223053	Infant	15	Apr 2016	Sep 2016	GP	Screening
33	224287	Infant	5	Jan/Feb 2016	Nov 2016	GP	Screening
34	225425	Grandparent	11	Not hospitalised	Nov 2016	GP	Screening
35	225423	Father	11	Not hospitalised	Dec 2016	GP	Screening
36	229989	Sibling	12	Not hospitalised	Feb 2017	GP	Screening

BCC: breast care centre; GP: general practitioner; HCF: other healthcare facility; HCW: healthcare worker; MRSA: meticillin-resistant *Staphylococcus aureus*; MU: maternity unit; NA: not applicable.

^a^ Outbreak cases were assigned case numbers according to the sampling date.

^b^ This includes all mothers, infants and staff members who had worked or stayed in the maternity unit between 1 January and 30 June 2016.

^c^ MRSA was isolated 3 months after travel to Thailand.

^d^ MRSA was subsequently isolated from a postoperative wound on 12 May 2016.

### Outbreak cases identified outside the maternity unit

Retrospective and prospective review of the LIVA database revealed that 30 other individuals outside the hospital had tested positive for MRSA CC398 of MALDI-TOF MS subgroup A8.3 between January 2016 and February 2017. All 30 cases had links to the maternity unit, including eight mothers and 13 infants who had stayed in the unit between 1 January and 9 May 2016 and nine household contacts (Figure 1 and Table 1). Notably, the LIVA database does not indicate whether mothers, infants and household contacts were tested because they were contacted by the local infection control team or if they were tested for other reasons.

### Outbreak description

In total, 36 MRSA cases with epidemiological links to the outbreak in the maternity unit were identified between 10 February 2016 and 22 February 2017. Of these, 27 (15 infants, 11 mothers and one HCW) had stayed in the maternity unit between 31 January and 10 May 2016 (Figure 1 and Table 1). The mean length of stay was 5.1 days (range: 2–11 days). Four of these infants (outbreak cases 3, 12, 14 and 18) had subsequently stayed in the neonatal unit, while one infant (outbreak case 2) had been visiting the neonatal unit’s outpatient clinic (Figure 1 and Table 1), but this did not lead to any secondary cases. Eighteen of the 36 possible outbreak cases (seven infants, nine mothers, one father and one sibling) were infected at the time of diagnosis (Figure 1 and Table 1). The majority of mothers had abscesses in the breast or axillary area, while pustulosis of the scalp and face predominated among infants. All MRSA isolates from the 36 outbreak cases had the same unique MALDI-TOF MS subgroup A8.3, belonged to CC398, had *spa* type t034 and carried *mecA*, *scn* and *lukF-PV*. The HCW was identified as the most likely source of the outbreak and was therefore relieved from duty for 3 days, during which the HCW began MRSA decolonisation therapy. By the end of May 2016, no more cases were detected in the maternity unit and the outbreak was declared over.

### Identification of cases from the national MRSA database

Review of the national MRSA database identified 37 cases that had no epidemiological links to the outbreak in the maternity unit, but whose isolates also belonged to CC398, had *spa* type t034 and carried *mecA*, *scn* and *lukF-PV* (Table 2). Twenty-nine of these were infected at the time of diagnosis, most of which presented in primary care with skin abscesses.

**Table 2:** Description of MRSA cases and isolates in the national MRSA database, Denmark, 2006–2017 (n = 37)

Isolate/case ID	Case description	Household cluster	Sampling date	Sampling place	Specimen source	Relation to south-eastern Asia	Lineage	Phylogenetic cluster
50148	Adult	1	Jan 2006	HCF	Skin abscess	Yes	L1	A
51306	Child	1	May 2006	HCF	Screening	Yes	L1	A
51726	Child	NA	Jun 2006	HCF	Skin abscess	Yes	L1	NA
55600	Child	1	Mar 2007	HCF	Wound infection	Yes	L1	A
55730	Child	1	Mar 2007	HCF	Screening	Yes	L1	A
160793	Adult	NA	Jan 2012	Unknown	Skin abscess	Yes	L2	NA
173861	Adult	2	May 2013	GP	Skin abscess	Yes	L2	B
174399	Adult	2	May 2013	Unknown	Screening	Yes	L2	B
176761	Adult	3	Aug 2013	HCF	Skin abscess	No	L2	C
176987	Adult	3	Sep 2013	GP	Screening	Unknown	L2	C
185025	Adult	NA	Mar 2014	GP	Skin abscess	Yes	L2	D
187597	Adult	NA	Jun 2014	GP	Screening	No	L2	D
194365	Adult	NA	Dec 2014	HCF	Skin abscess	Yes	L2	NA
203065	Child	NA	May 2015	GP	Wound infection	Unknown	L2	E
203343	Adult	NA	Jun 2015	GP	Skin abscess	Yes	L2	NA
205059	Adult	NA	Jul 2015	HCF	Skin abscess	Yes	L2	F
206889	Adult	4	Sep 2015	Unknown	Wound infection	No	L2	E
208403	Adult	4	Oct 2015	GP	Screening	No	L2	E
212591	Adult	NA	Jan 2016	GP	Wound infection	Yes	L2	NA
214119	Adult	6	Feb 2016	GP	Paronychia	No	L2	G
214133	Adult	NA	Jan 2016	GP	Skin abscess	No	L2	G
214137	Adult	6	Feb 2016	HCF	Skin abscess	No	L2	G
217191	Child	10	May 2016	GP	Screening	Yes	L2	H
218093	Adult	NA	May 2016	HCF	Skin abscess	Yes	L2	I
219061	Adult	NA	Jun 2016	GP	Wound infection	Yes	L2	I
219077	Adult	NA	Jun 2016	GP	Skin abscess	No	L2	I
219969	Adult	NA	Jun 2016	GP	Wound infection	No	L2	NA
220875	Adult	NA	Aug 2016	HCF	Wound infection	Yes	L2	NA
223021	Adult	NA	Sep 2016	GP	Wound infection	Yes	L2	NA
224479	Adult	10	Nov 2016	HCF	Skin abscess	Yes	L2	H
225753	Adult	NA	Aug 2016	HCF	Wound infection	Yes	L2 outbreak^b^	J
230011	Adult	NA	Feb 2017	GP	Skin abscess	No	L2	NA
230183	Adult	NA	Feb 2017	GP	Skin abscess	Yes	L2	NA
230425	Adult	16	Mar 2017	GP	Skin abscess	No	L2 outbreak^c^	J
231383	Child	NA	Mar 2017	GP	Skin abscess	No	L2	F
231391	Adult	NA	Apr 2017	Unknown	Skin abscess	No	L2	E
231535	Adult	16	Apr 2017	GP	Screening	No	L2 outbreak^d^	J

GP: general practitioner; HCF: other healthcare facility; MRSA: meticillin-resistant *Staphylococcus aureus;* NA: not applicable.

^a^ Relation to south-eastern Asia comprises cases who were either born in or had recently visited south-eastern Asia or had known contact with such individuals.

^b^ Outbreak case 37.

^c^ Outbreak case 38.

^d^ Outbreak case 39.

### Whole genome sequencing

**Figure 2:**
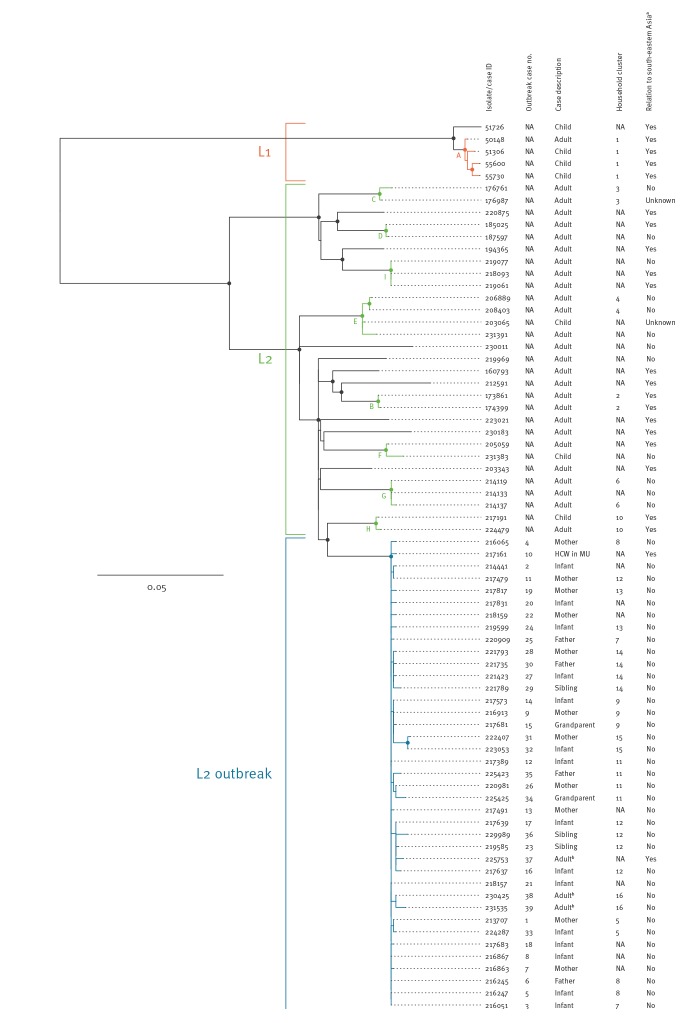
Maximum-likelihood phylogeny of Panton-Valentine leukocidin-positive meticillin-resistant *Staphylococcus aureus* clonal complex 398 isolates, Denmark, 2006–2017 (n = 73)

HCW: healthcare worker; MU: maternity unit; NA: not applicable.

^a^ Relation to south-eastern Asia comprises cases who were either born in or had recently visited south-eastern Asia or had known contact with such individuals.

^b^ The three community cases identified through review of the national MRSA database.

Analysis of whole genome sequence data from the 73 PVL-positive MRSA CC398 isolates, including 36 isolates from the outbreak investigation and 37 additional isolates from the national MRSA database, showed that they all contained the core SNPs used to differentiate the human variant of MRSA CC398 from the LA variant [11]. Mapping sequence reads for each isolate against the *S*. *aureus* CC398 reference isolate 71193 identified 1,071 high-quality core SNPs among the 73 isolates after recombination was removed from the SNP alignment. Phylogenetic analysis revealed two distinct lineages (L1 and L2) separated by 254–284 SNPs (Figure 2). L1 contained five isolates that differed by 5–22 SNPs, belonged to ST398, carried *spa* type t034, harboured a composite type Vb (5C2 and 5) SCC*mec* element and contained *erm*(C) conferring resistance to macrolides, lincosamides and streptogramin B (MLS_B_), as well as chromosomal mutations associated with resistance to quinolones (Ser84Leu in *gyrA* and Ser80Phe in *grlA*). L2 contained 68 isolates that differed by 0–148 SNPs, belonged to ST1232 and carried either *spa* type t034 (n = 66), t1255 (n = 1) or t1928 (n = 1). All L2 isolates carried a non-subtypable SCC*mec* V (5C2) element and contained *erm*(A), *spc* and *tet*(K) encoding resistance to MLS_B_, aminoglycosides (spectinomycin) and tetracyclines, respectively. In addition, four L2 isolates contained *aac*(6')-*aph*(2”) encoding resistance to aminoglycosides, one of which also carried *erm*(B) encoding resistance to MLS_B_. The 36 isolates considered part of the outbreak belonged to L2 and formed a well-supported cluster together with three isolates from community cases living in close proximity to Kolding Hospital. They are therefore referred to as outbreak cases 37, 38 and 39, respectively (Figure 2 and Table 2). The 39 L2 outbreak isolates differed from the L2 non-outbreak isolates by 47–140 SNPs and were more tightly clustered (0–15 SNPs vs 0–148 SNPs). Besides *erm*(A), *spc* and *tet*(K), which were present in all L2 isolates, the outbreak isolates also contained *dfrG* conferring resistance to trimethoprim.

The phylogeny was estimated from 1,071 high-quality core single nucleotide polymorphisms (SNPs) among the 73 isolates after recombination was removed from the SNP alignment, by using a general time reversible (GTR) model of nt substitution. Coloured branches indicate phylogenetic clusters (A-I) containing L1 and L2 isolates that differed by no more than 15 SNPs, which corresponds to the maximum number of pairwise SNP differences among the L2 outbreak isolates. Bootstrap values above 90% are illustrated by filled circles at the nodes. The scale bar represents the number of nt substitutions per variable site.

**Figure 3:**
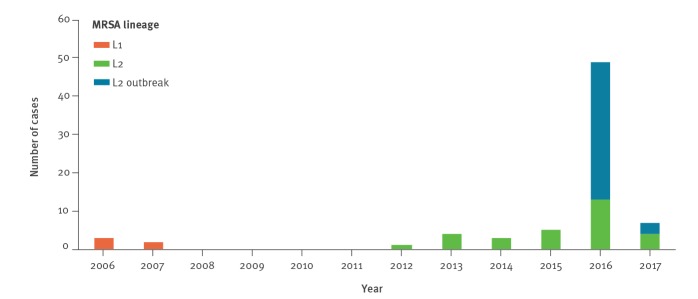
Temporal trend of Panton-Valentine leukocidin-positive meticillin-resistant *Staphylococcus aureus* clonal complex 398, Denmark, 2006–2017 (n = 73)

The temporal distribution of L1, L2 non-outbreak and L2 outbreak isolates is illustrated in Figure 3. The L1 isolates were detected in five cases from two households between 18 January 2006 and 15 March 2007, including four children adopted from south-eastern Asia and one of their parents (Figure 2 and Table 2). The L2 non-outbreak isolates were detected in 29 cases from 24 households between 16 January 2012 and 3 March 2017; 15 cases were either born in or had recently visited south-eastern Asia (i.e. Vietnam, Thailand or Cambodia) or had known contact with such individuals (Figure 2 and Table 2). We then divided the L1 and L2 non-outbreak isolates into clusters containing isolates that differed by no more than 15 SNPs, which corresponds to the maximum number of pairwise SNP differences among the L2 outbreak isolates. This led to the identification of nine phylogenetic clusters (A-I) involving four of five L1 isolates and 20 of 29 L2 non-outbreak isolates (Figure 2 and Table 2). L1 and L2 non-outbreak isolates from the same households always clustered together, supporting that transmission had occurred in at least six households. In addition, five clusters (D, E, F, G and I) contained L2 non-outbreak isolates from more than one household. Four of these clusters involved individuals with established or putative epidemiological links, including known community contacts (D and I), living in the same town (G) or living in the same municipality (E), while one cluster (F) involved people with no apparent epidemiological links. This indicates spread of L2 non-outbreak isolates from the households into the local community.

MRSA: meticillin-resistant *Staphylococcus aureus*.

The L2 outbreak strain was detected in 39 cases between 10 February 2016 and 5 April 2017, including 15 infants, 11 mothers, nine household contacts, one HCW and three community cases (Figure 1, Figure 2, Table 1, Table 2). Two of 39 outbreak cases had links to south-eastern Asia, including the HCW who had been on a 2-week holiday in Thailand some weeks before the outbreak and one community case who was Vietnamese. The community case lived ca 500 m from five of the outbreak cases. The isolate formed a well-supported cluster together with the outbreak isolates from the children, but not with that from the mother (Figure 2).

### Outbreak control measures

The HCW who screened positive for MRSA was promptly relieved from clinical duties for 3 days, during which the HCW began MRSA decolonisation therapy. Since the HCW had probably carried the outbreak strain since January 2016, a special team composed of members of the local infection control team and NRLAR epidemiologists was deployed to identify probable cases among mothers and infants who stayed in the maternity unit between 1 January and 9 May 2016 (i.e. before the outbreak investigation in the maternity unit was initiated) through retrospective and prospective review of local and national MRSA databases. In addition, all mothers who stayed in the maternity unit between 1 April and 9 May 2016 were contacted by letter and encouraged to undergo screening, along with their infants and household contacts. Decolonisation therapy was offered to all MRSA-positive individuals and their household contacts, in line with the Danish guidelines for the management of MRSA in healthcare settings [5].

## Discussion

This study describes an unusual MRSA outbreak in the maternity unit at Kolding Hospital. The outbreak strain was a PVL-positive human variant of MRSA CC398 of south-eastern Asian origin. The collaboration between the local infection control team and NRLAR, as well as the combined use of local and national surveillance data, extensive local screening of patients and hospital staff members, MALDI-TOF MS as a first-line tool for rapid subgrouping of MRSA isolates and whole genome sequencing made it possible to identify the source of the outbreak. These methods also made it possible to detect transmission events between patients and their household members, as well as subsequent spillover into the community.

In addition to the HCW, one of the community cases had links to south-eastern Asia and might have been in contact with five of the outbreak cases (a mother and her four children). However, we deemed it unlikely that the community case was the source of the outbreak, as the mother in question was admitted to the hospital on 2 May 2016, almost 3 months after the first outbreak case (Figure 1 and Table 1). In addition, the outbreak isolates were, on average, more closely related to the isolate from the HCW than to the isolate from the community case (4.4 SNPs vs 6.9 SNPs). Together, these findings support the notion that the HCW acquired the outbreak strain while in Thailand and introduced it into the hospital after the holiday. From there, it spread within the patients’ households and into the local community.

The PVL-positive human variant of MRSA CC398 has been recognised as a relatively frequent cause of infections among individuals in China, both in healthcare settings and in the community. In Europe, however, it has mainly been associated with carriage and infection in people with links to south-eastern Asia, including China, the Philippines, South Korea and Vietnam [7,12-14]. In addition, the type Vb (5C2 and 5) SCC*mec* element carried by L1 isolates is widely distributed among other community-associated MRSA clones in south-eastern Asia [15,16]. These findings support our assumption that the PVL-positive human variant of MRSA CC398 originates from south-eastern Asia.

Short hospital stays make it difficult to identify HAIs—let alone outbreaks of HAIs—because many cases experience disease onset while in the community. During the outbreak described here, most infections developed after discharge from hospital. In Denmark, the average number of bed-days per hospital admission was 2.91 in 2017 [17], leaving little time for a hospital-acquired MRSA strain to cause clinical symptoms of infection while a patient is hospitalised. This suggests that the annual numbers of hospital-acquired MRSA infections reported in DANMAP might be underestimated.

Previous studies have shown that skin and soft tissue infections (SSTIs) in travellers returning from high-prevalence countries are an important source of antibiotic-resistant *S*. *aureus*, including PVL-positive MRSA clones, and that nasal carriage may also play a role [14,18,19]. However, the Danish guidelines for the management of MRSA in healthcare settings only recommend screening HCWs and patients if they have had recent contact with a known MRSA carrier, stayed in a healthcare facility during an MRSA outbreak, been in a high-risk situation outside Denmark (e.g. hospitals, war zones, refugee camps or orphanages) or been in direct or indirect contact with pigs or mink [5]. The HCW, who is suspected to have acquired the outbreak strain while on holiday in Thailand, did not present with any of these risk factors. This suggests that the outbreak strain originated from a community rather than a healthcare reservoir in Thailand. Furthermore, the behaviour of the outbreak strain—with transmission between family members, onward spread into the surrounding community and a clinical picture dominated by SSTIs—resembles the epidemiology of other PVL-positive community-associated MRSA clones, such as CC30 and CC59 in Asia [16], CC80 in Europe [20] and US300 (CC8) in the United States [21] (see [22] also for a recent review on PVL-positive *S*. *aureus*). The emergence and rapid spread of community-associated MRSA clones in various parts of the world and the findings of this and other studies [23-25] suggest that international travellers could lead to import of community-associated MRSA and subsequent spread into hospitals. This notion is supported by the finding that imported MRSA clones accounted for an estimated 24% (325/1,356) of all MRSA infections in Denmark in 2016 [3].

### Lessons learnt

When the outbreak investigation started, it was not common practice for NRLAR to inform the local clinical microbiology departments about the presence or absence of PVL in submitted MRSA isolates. It is possible that the outbreak and its source could have been detected earlier if the local infection control team had been aware of the unusually high number of mothers and infants testing positive for the PVL-positive human variant of MRSA CC398 in February, March and April 2016. NRLAR has subsequently put this into practice and now informs general practitioners and local clinical microbiology departments of the presence or absence of *lukF-PV*, together with the *spa* type and/or the clonal complex.

The outbreak investigation might also have been more timely if there was an automatic outbreak detection system that could have linked the 21 mothers and infants who had been discharged before the start of the outbreak investigation. As no such system is in place, these cases were first identified during a retrospective review of the LIVA database in early May 2016. SSI has laid out plans for a national, real-time, electronic surveillance system based on data from MiBa, a nationwide, automatically updated database containing all clinical microbiology and strain typing results from hospitals, general practitioners and national reference laboratories [26], and MiBAlert, a warning system that generates an alert in the electronic medical records of individuals carrying multidrug-resistant bacteria [27]. This planned surveillance system will be a valuable tool for rapid outbreak detection across healthcare sectors and will overcome the obstacles involved in detecting cases outside hospital settings.

### Conclusions

This study describes the investigation of an unusual hospital outbreak caused by the PVL-positive human variant of MRSA CC398 of south-eastern Asian origin. The use of MALDI-TOF MS as a first-line tool for subgrouping of MRSA isolates allowed accurate case detection and identification of the MRSA CC398 outbreak. Whole genome sequencing facilitated detection of transmission pathways within and outside the hospital, and is now used routinely during outbreak investigations in Denmark. The data presented here and previous findings suggest that international travellers returning from high-prevalence countries could be an important source of PVL-positive community-associated MRSA. Identification and screening of individuals with a history of travel-related SSTI, particularly HCWs, may therefore be taken into consideration during the next evaluation of the Danish MRSA guidelines.
